# Effects of Lysulin™ supplementation on pre-diabetes: study protocol for a randomized controlled trial

**DOI:** 10.1186/s13063-019-3269-8

**Published:** 2019-03-18

**Authors:** Priyanga Ranasinghe, Ranil Jayawardena, Lal Chandrasena, Vivianne Noetzel, John Burd

**Affiliations:** 1 Department of Pharmacology, Faculty of Medicine, University of Colombo, Colombo, Sri Lanka; 2 Department of Physiology, Faculty of Medicine, University of Colombo, Colombo, Sri Lanka; 3 Institute of Health and Biomedical Innovation, Queensland University of Technology, Brisbane, QLD Australia; 4 Nawaloka Hospital Research and Education Foundation (NHREF), Nawaloka Hospitals PLC, Colombo, Sri Lanka; 5 Lysulin, Inc., San Diego, CA USA

**Keywords:** Lysulin™, Pre-diabetes, Lysine, Vitamin C, Zinc

## Abstract

**Background:**

Diabetes mellitus is rapidly becoming one of the leading causes of morbidity and mortality worldwide. Preventive measures have become important, especially in the South Asian region and other parts of the world where diabetes is becoming increasingly prevalent. We postulate that a product containing amino acid lysine, micronutrient zinc and vitamin C will have beneficial effects on glycemic control and disease progression in patients with pre-diabetes and we aim to evaluate this hypothesis using a phase II/III randomized double-blind controlled clinical trial design.

**Methods/design:**

The study will be conducted as a randomized, double-blind, placebo-controlled clinical trial for a period of 6 months. The study has been approved by the Ethics Review Committee of Faculty of Medicine, University of Colombo, Sri Lanka. A total of 110 adults with pre-diabetes will be recruited for the study. They will be randomly assigned to the test and placebo groups on a 1:1 ratio. The test group will receive two tablets of Lysulin™ three times a day and the control group will receive identical placebo tablets. The study drugs will be double blinded to both investigators and subjects. The visits and the evaluations will be done as follows: screening (visit 0), 1 month (visit 1), 3 months (visit 2) and 6 months (visit 4). The primary outcome will be defined as change in glycemic control measured by HbA1c from baseline. Data will be analyzed using SPSS v16.

**Discussion:**

To our knowledge this is one of the first randomized controlled trials evaluating the effects of Lysulin™ (a combination of lysine, vitamin C and zinc) in pre-diabetes. This study will provide the necessary groundwork for future large-scale multicentered clinical trials. The result, positive or negative, should provide a step change in the evidence guiding current and future policies regarding management of pre-diabetes.

**Trial registration:**

Sri Lanka Clinical Trials Registry, SLCTR/2018/022. Registered on 13 July 2018. Study protocol version 2.0 (23 March 2018).

**Electronic supplementary material:**

The online version of this article (10.1186/s13063-019-3269-8) contains supplementary material, which is available to authorized users.

## Background

Diabetes mellitus is rapidly becoming one of the leading causes of morbidity and mortality worldwide [1]. In most regions of the world, diabetes has reached epidemic proportions, and in the South Asian region alone the projected increase in prevalence of diabetes is out of proportion to the estimated population increase. For instance, the total population will increase by 40% from 2000 to 2030, but the percentage of change in prevalence of diabetes is estimated to be 151% [2]. This has placed an increasing financial burden on health care systems around the world, and in the USA alone the total estimated cost of diagnosed diabetes in 2017 is $327 billion, including $237 million in direct medical costs [3]. An estimated 80% of the world’s population with diabetes are from developing countries, overwhelming their existing health care facilities [1]. Therefore, preventive measures have become important, especially in the South Asian region and other parts of the world where diabetes is becoming increasingly prevalent.

The causes of type 2 diabetes are multifactorial, and diet plays an important role in its incidence, severity and management [4]. Recent studies have demonstrated that numerous herbal and nutraceutical products have beneficial effects in patients by improving glucose and lipid metabolism, antioxidant status, disease progression and capillary function [5]. Lysulin™ is one such nutraceutical tablet manufactured in the USA which contains the essential amino acid lysine, micronutrient zinc and vitamin C as the active ingredients, together with other standard excipients (Lysulin Inc., San Diego, CA, USA) [6]. Lysulin™ is considered a dietary supplement under the Dietary Supplement Health and Education Act of 1994 of the US National Institute of Health (NIH) Food and Drug Regulatory Authority (FDA), as it contains only amino acids, vitamins and minerals [7].

Lysine is an essential amino acid that plays a major role in calcium absorption, building muscle protein, and the body’s production of hormones, enzymes and antibodies. It has also shown numerous beneficial effects in the treatment/prevention of diabetes and/or its complications in in-vivo animal and human studies. In diabetes-induced animal models, lysine has shown beneficial effect in lowering blood glucose as well as acting as an inhibitor of protein glycation [8]. Furthermore, the ability of lysine to reduce the formation of glycated proteins in diabetes-induced rats has also been shown to delay the appearance of the late pathologies associated with protein glycation [9]. Lysine is known to react with glucose, with the glycated amino acid being excreted in urine, and it has been shown to markedly attenuate the glucose response to ingested glucose without a change in insulin response in humans [10]. Furthermore, studies have shown that lysine reduces the formation of glycated proteins in diabetes-induced animal models [8]. Glycated proteins are known to be involved in the pathogenesis of several chronic diabetes complications, including nephropathy leading to chronic kidney disease, neuropathy and retinopathy, as well as in other macrovascular complications [11–13]. Hence, it is evident that lysine may have potentially beneficial effect on the reduction of blood glucose as well as on the progression of diabetes and its complications.

Zinc is involved in numerous metabolic pathways as a cofactor for more than 300 enzymes [7]. Insulin, which contains a variable number of zinc atoms, is stored in β-cells of the pancreas and released into the portal venous system at the time of β-cell degranulation. Studies have shown that zinc plays an important role for insulin action, carbohydrate and protein metabolism [10]. Zinc absorption is also known to be altered in patients with diabetes [14]. Numerous studies have shown that zinc supplementation improves glycemic control in patients with type 2 diabetes, with a resultant reduction in HbA1c of around 0.5% in pooled analysis [15]. A study conducted in Bangladesh demonstrated that individuals with pre-diabetes are also known to have lower serum zinc concentrations compared to those who are healthy [16]. Furthermore, a recently concluded clinical trial on patients with pre-diabetes demonstrated that zinc supplementation helps to reduce blood glucose and insulin resistance, while improving β-cell function [17]. Furthermore, disease progression to diabetes was also reduced and beneficial effects of supplementation were also noted on total and LDL cholesterol [17].

Ascorbic acid (vitamin C), an antioxidant vitamin, plays an important role in protecting free radical-induced damage. Previous study has shown a decrease in the basal vitamin C level in type 2 diabetes [18]. Vitamin C is structurally similar to glucose and can replace it in many chemical reactions, and thus is effective for prevention of nonenzymatic glycosylation of protein [19]. Furthermore, randomized controlled trials have shown that supplementation with vitamin C reduces blood glucose and serum lipids and improves HbA1c in type 2 diabetes [20]. Hence, we postulate that a product containing amino acid lysine, micronutrient zinc and vitamin C will have beneficial effects on glycemic control and disease progression in patients with pre-diabetes. The present study aims to evaluate this hypothesis using a phase II/III randomized double-blind controlled clinical trial design.

## Methods/design

**Fig. 1:**
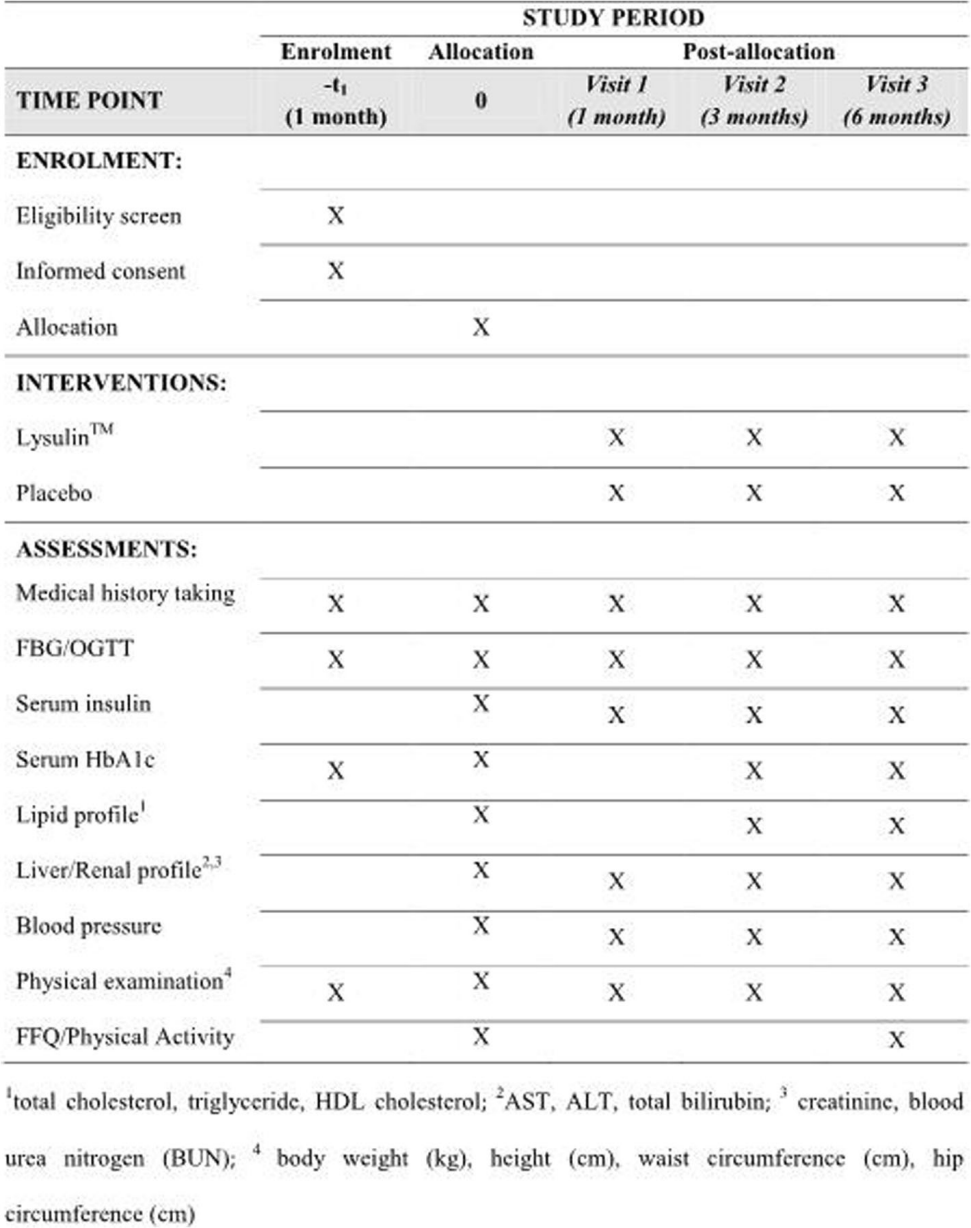
Summarized study schedule of enrolment, interventions and assessments in the clinical trial. ALT alanine aminotransferase, AST aspartate aminotransferase, FBG fasting blood glucose, FFQ Food Frequency Questionnaire, HDL high-density lipoprotein, OGTT oral glucose tolerance test

This protocol was written following the Standard Protocol Items: Recommendations for Interventional trials (SPIRIT) checklist (see Additional file 1) [21]. The schedule of trial enrollment (SPIRIT figure), interventions and assessments is presented in Fig. 1.

### Objectives and hypothesis

#### Hypotheses

- Primary—the glycemic control of people with pre-diabetes who are treated with Lysulin™ tablets will be better than that of the control group.
- Secondary—progression to diabetes would be reduced and other related metabolic parameters (serum insulin, total cholesterol, triglyceride, HDL cholesterol, LDL cholesterol and blood pressure) will be improved in the treatment group in comparison to the control group.

#### Objectives

- Primary—to evaluate the potential effects of Lysulin™ tablet supplementation on glycemic control and disease progression in patients with pre-diabetes.
- Secondary—to evaluate effects on serum lipids, anthropometric parameters, blood pressure and appetite, and also to study potential effects of regular administration of Lysulin™ tablets on liver and kidney function and the occurrence of self-reported side effects in patients with pre-diabetes.

### Study design

The study will be a phase II/III randomized, double-blind, placebo-controlled clinical trial. It will be conducted at the Nawaloka Hospital Research and Education Foundation (NHREF), Nawaloka Hospitals PLC, Colombo, Sri Lanka for a period of 6 months, assessing the effects of daily supplementation of Lysulin™ tablets in patients with pre-diabetes.

### Sample size

A total of 110 adults with pre-diabetes will be recruited for the study after a screening test confirming the presence of pre-diabetes as defined in ‘Population’ and further eligibility screening against other inclusion/exclusion criteria described in ‘Inclusion and exclusion criteria’. Summaries of previous studies of oral antidiabetic drugs suggest that they reduce HbA1a levels by 0.5–1.5% [22]. Numerous studies have shown that zinc supplementation reduces HbA1c by around 0.5% [15]. Hence, in the absence of preliminary studies in a comparable cohort, we assumed an HbA1c reduction of 0.5%. The number of patients required for determination of a 0.5% reduction of HbA1c in the treatment arm, in comparison with the placebo arm, at 80% power and 95% confidence interval with a drop-out rate of 20% was 108 patients. Hence, a total of 110 adults with diagnosed pre-diabetes will be recruited for the study. Subjects will be randomly and equally assigned into two groups (*n* = 55 each) and will receive either Lysulin™ oral tablet or an identical placebo daily for a period of 6 months. The formula used for sample size calculation was as follows:

$$ N=\left[\left(1/{q}_1+1/{q}_2\right)\times {\left({Z}_{\alpha }+{Z}_{\beta}\right)}^2\right]/\left[{\left(E/S\right)}^2\right], $$

where *N* = sample size,

*q*_1_ = proportion of subjects in the treatment group (0.5),

*q*_2_ = proportion of subjects in the placebo group (0.5),

*Z*_*α*_ = critical value of the normal distribution at *α* (*α* = 0.05 and critical value = 1.96),

*Z*_*β*_ = critical value of the normal distribution at *β* (*β* = 0.2 and critical value = 0.84),

*E* = effect size (0.5% difference in HbA1c between the two groups) and

*S* = standard deviation of HbA1c in the population (in the absence of previous published studies on pre-diabetes in Sri Lanka that reported HbA1c values, this was assumed to be 0.82%, based on previous research conducted among patients with diabetes in Sri Lanka) [23].

### Population

Pre-diabetes is defined as the presence of fasting plasma glucose (FPG) between 100 and 125 mg/dl (impaired fasting glucose (IFG)), or 2-h post oral glucose tolerance test (OGTT) plasma glucose between 140 and 199 mg/dl (impaired glucose tolerance (IGT)), or both IFG and IGT, or an HbA1c value between 5.7 and 6.4% [24].

### Inclusion and exclusion criteria

#### Inclusion criteria

- Age between 18 and 60 years.
- Screening test confirming the presence of pre-diabetes as defined in ‘Population’.

#### Exclusion criteria

- On any other vitamin or mineral supplementations.
- Current use of a weight loss medicine or dietary modification.
- History of diabetes mellitus.
- Presently having acute diseases or other untreated illness requiring treatment.
- Impaired hepatic or renal functions.
- Patients with any malignancy or any other unrelated chronic illness.
- Patients with cardiac, liver or respiratory failure.
- Any condition in the opinion of the primary investigator that would contraindicate the patient’s participation.
- Allergy to any of the constituents of the tablets.
- Lactation, pregnancy or unwillingness to use an effective form of birth control for women of childbearing age.

#### Suspension criteria

- Subject’s demand to discontinue the study.
- Serious adverse events or unusual changes in clinical test results.
- Principal investigator’s decision to terminate the study (low rates of compliance, complications or unable to sustain the study for various reasons).

### Randomization

Randomization for the parallel treatment arms will be carried out after checking the inclusion and exclusion criteria. The patients will be randomized in a 1:1 ratio according to the method of block randomization with a block size of 4. The population will be stratified at randomization based on age (< 30 years and ≥ 30 years) and gender to ensure equal distribution of these variables in the two arms. The randomization sequence would be generated using the SPSS statistical software package (version 14.0). Concealment of the allocation sequences will be done using sequentially numbered, opaque, sealed, color-coded envelopes. Eligibility assessment and enrolment will be done by one independent investigator, while another investigator will be involved in randomization. The investigator involved in randomization shall open the next respective color-coded envelope depending on the stratification and allocate the patient to the respective group (treatment or control). The treatment and control groups shall be designated as either ‘A’ or ‘B’ and this allocation shall be kept in a sealed envelope in a secure place during the course of the study. The manufacturer will be responsible for preparing the study medications designated either ‘A’ or ‘B’.

### Blinding

The investigators and patients would be blind to the treatment allocations. The medication will be delivered in similar packets and labels with its own sequence number. The allocation sequence number will be generated by one of the investigators not involved in managing patients. Envelopes containing a monthly supply containing Lysulin™ tablets or placebo will be prepared according to the randomization sequence and supplied to the patients by the principal investigator or research assistants when they are randomized to the trial.

### Interventions

The treatment drug will be a tablet containing amino acid lysine, elemental zinc and vitamin C as the active ingredient. All of the active ingredients are supplemented within the Recommended Daily Allowance (RDA) [25, 26]. The placebo tablet contains inactive ingredients (magnesium stearate and microcrystalline cellulose) and will be manufactured to have a similar appearance, shape, weight, taste, color, smell and texture. The drug manufacturing will be done at a US FDA-registered and GMP-certified manufacturing site in the USA. The manufacturer will be responsible for supplying the Lysulin™ and placebo tablets. However, the manufacturer/funding organization shall not be involved in any aspects related to the conduct of the study, including patient recruitment, follow-up and analysis of data.

Recruited subjects will receive either two tablets of Lysulin™ or placebo three times daily, taken 1 h before meals for a period of 6 months. Envelopes containing a monthly supply of Lysulin™ or placebo tablet will be prepared according to the randomization sequence and supplied to the patients. Participants in both groups will receive uniform advice about diet and physical activity, which are considered to be potential confounder variables affecting glycemic control.

### Study groups

The treatment group will receive Lysulin™ tablets (*n* = 55). The control group will receive placebo tablets (*n* = 55).

### Study period

The study will be for a period of 6 months and the visits and evaluations will be done as follows; screening (visit 0), 1 month (visit 1), 3 months (visit 2) and 6 months (visit 3) (Fig. 1).

### Outcomes

#### Primary outcome index

The primary outcome will be defined as the change in glycemic control measured by HbA1c from baseline.

#### Secondary outcomes

- Change in FPG and OGTT plasma glucose from baseline.
- Development of diabetes during follow-up, as indicated by either FPG > 125 mg/dl and/or 2-h OGTT plasma glucose > 199 mg/dl and/or HbA1c > 6.5%, confirmed during follow-up visits [27].
- Change in lipid profile from baseline (total cholesterol, LDL, HDL and TAG).
- Change in insulin resistance from baseline. Insulin resistance would be measured by the Homeostasis Model of Assessment—Insulin Resistance (HOMA-IR) calculations based on fasting blood glucose and fasting serum insulin. Β-cell function would be evaluated in vivo (HOMA-B calculation) and in vitro.
- Change in anthropometric assessment such as body weight, height, body mass index (BMI), waist circumference (WC), hip circumference (HC) and waist:hip ratio (WHR) from baseline.
- Change in systolic blood pressure (SBP) and diastolic blood pressure (DBP) from baseline.

Level of physical activity (International Physical Activity Questionnaire (IPAQ) short form) and dietary intake (validated Food Frequency Questionnaire) would be assessed in both groups as they are confounding factors affecting glycemic control.

#### Safety assessment index

The following information will be recorded/measured for the safety assessment; vital signs, general medical examinations, renal function tests, liver function tests and adverse events.

### Procedures

#### Recruitment

Participants will be recruited on a voluntary basis from a cohort of patients with pre-diabetes attending Nawaloka Hospitals PLC, Colombo, Sri Lanka. Informed written consent will be obtained from all study participants.

#### Study schedule

Fasting blood glucose, 2-h OGTT blood glucose, serum insulin, the liver profile (AST, ALT, PT/INR) and the renal profile (blood urea and serum creatinine) will be measured at the 0,1, 3 and 6 months. In addition, the serum lipid profile (total cholesterol, triglycerides, LDL cholesterol, HDL cholesterol) and serum HbA_1_c would be measured at baseline, at 3 months and on completion of the study. The details of items which will be measured at every visit are described in Fig. 1.

### Measurement tools

#### Anthropometric measurements

Height will be measured using Harpenden pocket stadiometers (Chasmors Ltd, London, UK) to the nearest 0.1 cm. Body weight will be measured in indoor light clothing to the nearest 0 .1 kg using a SALTER 920 digital weighing scale (Salter Ltd, Tonbridge, UK). Waist circumference will be measured midway between the iliac crest and the lower rib margin at the end of normal expiration using a plastic flexible tape to the nearest 0 .1 cm. Similarly, the hip circumference will be measured at the widest part of the buttocks in the inter-trochantric level to the nearest 0 .1 cm. All anthropometric measurements will be made by trained personnel. All anthropometric measurements (height, weight, BMI, WC, HC and WHR) will be made using standard calibrated equipment and following recommended guidelines [28]. The equipment used for anthropometric measurements will be calibrated by the Department of Measurement Units, Standards and Services, Colombo, Sri Lanka [29].

#### Dietary measurements

A culturally validated Food Frequency Questionnaire (FFQ) will be used to obtain habitual intake of calories, macronutrients and micronutrients [30]. The FFQ contains color photographs of three different portion sizes of four commonly consumed foods (rice, green vegetable curry, lentil curry and chicken) and a list of food items (*n* = 85) with their portion sizes and frequencies. Each respondent is expected to report consumption of each food according to frequency. Food items are categorized into eight food groups, namely: cereals; vegetables; pulses; meat; fruits; drinks; miscellaneous; and alcohol. The FFQ is interviewer-administered in the local language (Sinhalese and Tamil). The participants will be asked to recall their usual portion size and intake of foods listed within the FFQ over the past month. FFQ data will subsequently be analyzed and be reported as the intake of calories, carbohydrates, fat, proteins and dietary fibers per day. Furthermore, intake of lysine, vitamin C and zinc will also be analyzed.

#### Physical activity

Physical activity during the past week will be assessed using the translated and validated short version of the International Physical Activity Questionnaire (IPAQ) administered by an interviewer and will be presented as energy expenditure expressed in metabolic equivalent (MET) minutes/week [31]. The continuous score allows the estimation of the weekly energy expenditure expressed in MET minutes/week. This is obtained by multiplying the value of energy expenditure for the given physical activity in MET by the weekly frequency (days per week) and the time in minutes (minutes per day). These values are separately computed for the three domains of walking, moderate-intensity activities and vigorous-intensity activities using the following formulas:

$$ \mathrm{Walking}\ \mathrm{MET}\ \mathrm{minutes}/\mathrm{week}=3.3\times \mathrm{minutes}\times \mathrm{days}, $$

$$ \mathrm{Moderate}\ \mathrm{MET}\ \mathrm{minutes}/\mathrm{week}=4.0\times \mathrm{minutes}\times \mathrm{days}, $$

$$ \mathrm{Vigorous}\ \mathrm{MET}\ \mathrm{minutes}/\mathrm{week}=8.0\times \mathrm{minutes}\times \mathrm{days}. $$

A combined total physical activity MET minutes/week can be computed as the sum of these three domains.

#### Biochemical analysis

Centrifuged serum separated samples (2200–2500 rpm) will be used for the assays. The glucose assay will be performed by an enzymatic colorimetric (glucose oxidase) method in an RxDaytona™ chemical analyzer (Randox Laboratories Ltd, Antrim, UK). Total cholesterol and LDL cholesterol will be analyzed by an enzymatic colorimetric method in a Mindray BA-88A semi auto-analyzer (Mindray Medical International Ltd, China). HDL cholesterol will be determined by a precipitation method in a Mindray BA-88A semi auto-analyzer. Triglyceride will be analyzed by an enzymatic colorimetric method in a Mindray BA-88A semi auto-analyzer using commercially available enzymatic colorimetric determination kits for triglycerides.

#### Clinical examination

Seated blood pressure will be recorded on two occasions after at least a 10-min rest using an Accoson mercury sphygmomanometer (Accoson Healthcare, Asia-Pacific Region, Singapore).

#### Other

In addition to the aforementioned sociodemographic data, drugs and data on side effects would also be gathered via a questionnaire.

### Compliance calculation

Subjects will be asked to return remaining drugs and their compliance will be evaluated using the following formula:

$$ \mathrm{Compliance}\ \left(\%\right)=\left[\left(\mathrm{distributed}\ \mathrm{drugs}-\mathrm{remaining}\ \mathrm{drugs}\right)/\mathrm{distributed}\ \mathrm{drugs}\right]\times 100. $$

### Statistical analysis

Parametric and nonparametric statistical tests will be applied using SPSS version 16 (SPSS Inc., Chicago, IL, USA) and Stata/SE 10.0 (Stata Corporation, College Station, TX, USA) for data analysis. FFQ data will be analyzed using NutriSurvey 2007 (EBISpro, Germany), nutrient analysis software for Windows, modified for Sri Lankan food items and recipes, and will be reported as the intake of calories, carbohydrates, fat, proteins and dietary fibers per day. Summary statistics will be calculated and presented as mean, standard deviation and proportion by group. The baseline and end of study characteristics as well as the laboratory findings of the groups will be compared using two-sample and paired *t* test, and *P* < 0.05 will be considered significant. The Homeostasis Model Assessment (HOMA2) calculator will be used to calculate β-cell function (HOMA-β) and insulin resistance (HOMA-IR) based on fasting insulin and plasma glucose [32]. The Homeostatic Model Assessment (HOMA) is a method used to quantify insulin resistance and β-cell function. It has good correlation with the traditional, invasive and time-consuming euglycemic clamp method (*r* = 0.88) which is used to measure insulin resistance and β-cell function.

Multiple regression analyses will be performed, where change in FPG/OGTT/HbA1c post intervention from baseline are the continuous dependent variables and independent variables will be age (continuous), treatment group (0, placebo; 1, Lysulin™), gender (0, female; 1, male), physical activity (continuous), BMI (continuous), energy intake (continuous), carbohydrate intake (continuous) and baseline FPG/OGTT/HbA1c. All analyses will follow intention-to-treat principles and a prespecified analysis plan. Where appropriate, sensitivity analyses will be conducted (e.g., control for additional covariates; and bootstrapped *P* values for skewed outcomes). In the case of missing data values, we will apply mean imputation and regression imputation where rates are low, and consider multiple imputations where they exceed 10%.

### Adverse effect evaluation

All of the active ingredients are supplemented within the Recommended Daily Allowance (RDA) [25, 26]. Therefore, the type of adverse events expected are likely to be minor in nature. However, in the event of a probable adverse reaction the following precautions would ensure timely identification and management of patients:

- Reporting—mechanisms would be set up to ensure direct reporting of probable adverse events to investigators by patients (via telephone available 24 h on all days).
- During follow-up visits, probable adverse events would be noted by history and examination and investigated in detail. All adverse effects observed will be documented in the case report form (CRF).
- Any serious adverse event as defined in GCP guidelines will be reported to the National ADR monitoring center at the Department of Pharmacology, Faculty of Medicine, University of Colombo within 24 h, the Ethics Committee, Faculty of Medicine, University of Colombo and the Clinical Trials subcommittee of the Drug Regulatory Authority within 1 week.
- A Data Safety Monitoring Board (DSMB) identified by the investigators will evaluate all adverse events at regular intervals.
- Investigations—liver and renal functions would be assessed as detailed earlier.
- Management—in the event of an adverse reaction requiring in-hospital management, the facilities and expert management would be available at the Nawaloka Hospital PLC, Colombo, Sri Lanka.
- Termination of study—the complete clinical trial will be terminated prematurely if there is evidence that the safety of the trial participants can no longer be assured or new scientific information arises during course of the clinical trial regarding safety of the patients.

### Data collection

After filling out the case report form (CRF), data collection will be performed according to the standard operating procedures (SOPs) by the trained clinical research associates (CRAs).

### Data management and monitoring

#### Storage

Data will be entered by a minimum number of dedicated staff and saved in a dedicated computer with password protection. Samples would be stored in a secure facility, with redundant measures to ensure specimens are kept in compliant conditions at all times when in storage. Expert staff who have been trained specifically in sample storage and transportation would ensure all regulatory issues are properly handled. Storage technologies with the capability of monitoring the temperature of samples around the clock would be utilized.

#### Sample disposal

After each analysis is completed and with the approval of the Principal Investigator, the samples stored in the storage facility may be disposed of by the sample custodian. A Sample Disposal Sheet is completed and kept for further reference.

### Dissemination of study finding

The results of the study will be published in local and international peer-reviewed journals and presented at international conferences and clinical meetings.

### Ethical considerations

The study has been approved by the Ethics Review Committee of Faculty of Medicine, University of Colombo, Sri Lanka (EC/18/020). The trial is also registered at the Sri Lanka Clinical Trials Registry (SLCTR/18/022). The study will be conducted in compliance with the Declaration of Helsinki and the Good Clinical Practice (GCP) guidelines. Any change in trial protocol will be notified to the relevant regulatory authorities and trial participants, with new consent being taken from participants, if required.

## Discussion

In this article we describe the protocol for a clinical trial design evaluating the effects of Lysulin™ tablets in patients with pre-diabetes. To our knowledge this is one of the first randomized controlled trials evaluating the effects of supplementation of lysine, zinc and vitamin C in patients with pre-diabetes. Hence, the present study will provide the required foundation for future large-scale multicentered clinical trials. Given the present interest in using a variety of nutritional supplements to enhance glycemic control and reduce progression in patients with pre-diabetes, properly planned and methodical scientific studies are a timely necessity. However, in relation to lysine, zinc and vitamin C, presently there are no well-designed randomized control trials conducted for a satisfactory time period to support/refute this argument. Hence, the result of the present study, positive or negative, should provide a step change in the evidence guiding current and future policies regarding the use of Lysulin™ as a dietary supplementation and/or treatment in patients with pre-diabetes.

## Trial status

Enrollment for the trial has not yet started.

## Additional file

Additional file 1: SPIRIT 2013 checklist: recommended items to address in a clinical trial protocol and related documents. (DOC 122 kb)

## Abbreviations

ALT: Alanine aminotransferase
AST: Aspartate aminotransferase
CRA: Clinical research associate
CRF: Case report form
HDL: High-density lipoprotein
HOMA-IR: Homeostasis Model of Assessment—Insulin Resistance
IPAQ: International Physical Activity Questionnaire
LDL: Low-density lipoprotein
OGTT: Oral glucose tolerance test
SOP: Standard operating procedure
SPSS: Statistical Package for the Social Sciences
